# “They’ll be the ones that’s looking after it” - Unravelling institutional factors that shape children’s participation in urban planning for informal settlements

**DOI:** 10.1080/14733285.2022.2159331

**Published:** 2022-12-28

**Authors:** Robyn G. Mansfield

**Affiliations:** Monash Sustainable Development Institute, Monash University, Australia

**Keywords:** Sustainable development, SDG11, children’s participation, urban planning, informal settlements, water infrastructure

## Abstract

Sustainable Development Goal target 11.3 calls for ‘inclusive and sustainable urbanization and capacities for participatory, integrated and sustainable human settlement planning and management in all countries’, yet children are systematically excluded from decision-making in urban planning structures, particularly in vulnerable settings. This case study examines the factors that shape children’s participation in urban planning processes for the revitalisation of water infrastructure in the Revitalising Informal Settlements and their Environments (RISE) program in Suva, Fiji. This study aims to answer the research question ‘What are the factors that shape how children participate in informal settlement revitalisation in Suva, Fiji?’ The study utilises a qualitative case study to investigate the factors that underpin and reinforce structural, political, and economic systems of children’s exclusion. Qualitative interviews were conducted with 32 RISE staff and supporters to examine the individual, organisational and societal factors that shape children’s participation in the revitalisation of water infrastructure across 12 informal settlements. First, a typology of children’s participation is identified using Pells’ (2010) definitions of children’s participation as a foundation. Thornton, Ocasio and Lounsbury’s (2012) institutional logics framework is then used as a conceptual lens to develop a children’s participation logic for RISE. The findings contribute to discourse on children’s participation in the context of urban planning in informal settlements and critically examines the barriers that perpetuate exclusion of children from these processes.

## Introduction

Children continue to be excluded from participating in urban planning decision-making processes due to adult practices, attitudes, legal, socio-economic constraints, the political environment and cultural factors (UN Committee on the Rights of the Child (CRC) 2009). Of particular concern to the United Nations is the impact of compounding crises that further marginalise vulnerable populations, such as those living in settlements most susceptible to disasters, people living in urban low-income and informal settlements, and small island developing States (UN Secretary General 2021). As children are identified as the most vulnerable population group whose health and wellbeing is particularly impacted by their urban environment, understanding the barriers to mainstreaming their participation in urban planning processes for vulnerable settings is critical.

There are a number of international legal and policy frameworks that are particularly relevant in this context. Article 12 of the Convention on the Rights of the Child (CRC) is particularly important for this research due to its focus on children’s participation in all matters affecting them and has been ratified by all countries except the United States of America (United Nations 2021), entering all other States into a legally binding treaty supporting the individual human rights of children. (UN General Assembly 1989). The mechanism for hearing and taking children’s views into account is predominantly conceived through a participation lens where children can develop relationships with decision-makers and shape outcomes (UN Committee on the Rights of the Child (CRC) 2009; UN General Assembly 2002). In 2019 the United Nations General Assembly passed a resolution to gear up for a decade of action to achieve the Sustainable Development Goals, reinforcing the necessity for realising children’s rights and supporting their empowerment and their role as activists for creating a better world by arguing for the removal of all barriers to their participation (UN General Assembly 2019). The Sustainable Development Goal 11 (SDG11) ‘Make cities and human settlements inclusive, safe, resilient and sustainable’ makes several references to children and includes target 11.3 which calls for ‘inclusive and sustainable urbanization and capacities for participatory, integrated and sustainable human settlement planning and management in all countries’ (UN General Assembly 2017a). The New Urban Agenda reinforces the need for children’s participation in urban and territorial development (UN General Assembly 2016, Cl.148).

This study aims to answer the research question ‘What are the factors that shape how children participate in informal settlement revitalisation in Suva, Fiji?’ by investigating the factors that underpin and reinforce structural, political, and economic systems of children’s exclusion. This is done by examining a case study on children’s participation in a vulnerable setting, where urban planning processes are occurring. The Revitalising Informal Settlements and their Environments program (RISE) is led by the Monash Sustainable Development Institute and is trialling codesigned, water-sensitive and site-specific water and sanitation infrastructure across 12 informal settlements in Fiji and Indonesia through a randomised control study (RISE 2017b)^i^. RISE is responding to multiple crises of poor health of children, low-income stress, and water-related hazards. This study focuses on the Fiji settlements using qualitative research methods to interview decision-makers in the program to identify the factors that impact children’s participation. Interviewees’ understanding of children’s participation is first developed into a typology of participation using Pells’ (2010) definitions as a foundation. These factors are then analysed using Thornton, Ocasio and Lounsbury’s (2012) institutional logics framework to better understand what shapes the conscious and sub-conscious behaviours of individual actions and how.

## 1 Background

### 1.1 What is ‘participation’?

The United Nations Committee on the Rights of the Child (2009) states that ‘participation’ has become the accepted term to conceptualise Article 12 of the Convention on the Rights of the Child: ‘The Right of the Child to be Heard’. It defines children’s participation as expressing their views for consideration in decision-making, policy- and law-making through ongoing exchange in all relevant contexts of their lives (UN Committee on the Rights of the Child (CRC) 2009). The definition is broad and implementation problematic.

Mason and Bolzan (2009) note that the concept of participation itself, particularly a rights-based approach to participation, can be construed as informed by ‘Western liberalist’ values which is potentially incompatible with, or even disruptive to, social orders in different cultural contexts. Malone and Hartung (2010) further critique the language of participation as a narrow concept that sits within an adult-centred and driven structure that has failed to cascade into influencing decision-making. They argue that the field of children’s participation is dominated by empirical examples without a clear understanding of the purpose and the subsequent implications. They note that this has led to a proliferation of formally constructed, adult-initiated, manuals of practical tools, limiting children’s ability to participate in an authentic manner perpetuated through exploitative formal processes (Malone and Hartung 2010). Ultimately, they call for theorising of children’s environments in a manner where the system itself changes to include “all the possibilities of children’s participation” (Malone & Hartung 2010, 36).

A framework that has “relentlessly” dominated discourse and action on child participation across many disciplines (Malone and Hartung 2010) is Roger Hart’s ‘ladder of children’s participation’. The ladder was originally developed to support the implementation of the Convention on the Rights of the Child (Hart 1997). Hart’s ladder of children’s participation introduces a hierarchy of participation from ‘manipulation’ at the bottom of the ladder through tiered principles to ‘child initiated, shared decisions with adults’ at the top (Hart 1997). Hart himself has stepped back from this framework, arguing that its original purpose was to stimulate thinking and that the use of it in practice has neglected the complex and often ambiguous reality of participation (Hart 2008). Hart notes that the ladder has inadvertently developed into a hierarchical evaluative model of participation based on ‘Western’ theories of childhood, forcing children into participating at levels of power that aren’t necessarily personally or culturally supported (Hart 2008). Criticisms notwithstanding, the popularity of the ladder has not, however, resulted in mainstreaming of children’s participation. As a result of these concerns, Malone and Hartung (2010) highlight the need for research into how and why children participate and the implications of their participation, particularly within the context of localised culture as highlighted by Mason and Bolzan (2009).

Within this context, Pells (2010) has researched how children themselves identify with the right to participation in Rwanda, where the government has enshrined the CRC in national legislation. Pells conducted interviews and focus groups with children and youth to understand how the participatory principles of Article 12 have been operationalised within three non-governmental organisations (NGOs) in Rwanda, the barriers to youth participation and children’s perspective of participation (Pells 2010). Pells (2010) notes that children identify their right to participate even when this is contradictory to their cultural and social systems, and that their view of participation differs to that of adults. Pells (2010) offers a definition of participation created by children which frames participation as either ‘performed’, developed from children perceiving formal activities that are ‘extraordinary to their daily lives’, or as ‘lived’

relating to participation as ongoing, embedded in daily lives and manifesting as supportive relationships. While the study noted that ‘performed participation’ still had value, this was perceived to be manipulative or misleading and children advocated for ‘lived participation’ (Pells 2010).

While the study in this article focuses on adult subjects, Pells’ (2010) definitions of ‘performed’ and ‘lived’ participation have been used and expanded upon to capture participatory experiences that may be valued by children but not recognised by adults. This is essential for ensuring that this research does not contribute to the perpetuation of types of participation that may be viewed by children as exploitative and burdensome and to contribute to a greater understanding of the breadth of children’s participation.

### 1.2 Conceptual framework: Institutional logics

The above discussion suggests a need to comprehensively explore the processes and structures involved in children’s participation. This study mobilises Thornton, Ocasio and Lounsbury’s (2012) institutional logics framework in response to this need as an appropriate conceptual lens, and as a way of analysing the dynamics within those structures and processes to understand what is going on with children’s participation. Institutional logics theory has been recognised to be particularly useful for understanding structural and procedural barriers and enablers shaping individual and organisational behaviour at micro- (individual), meso- (organisational) and macro- (societal) levels (ibid).

The institutional logics framework has been used to examine influences on a number of professional fields and sectors and is more commonly used to determine changes to sectors over a historical timeframe such as historical analyses of the architecture, publishing and accounting sectors (Thornton, Jones and Kury 2005) and the Australian urban water sector (Fuenfschilling and Truffer 2014). Reay and Jones (2016) further legitimise using institutional logics in their methods for qualitative techniques for capturing logics through an inductive approach where qualitative interviews are categorised into patterns or sets of behaviours that align with single or multiple logics. Reay and Jones (2016) usefully reference examples that grapple with multiple logics and transformative processes, which align with the aims of this study in both capturing the existing logics and determining opportunities for transforming emerging logics to better support participation.

Winstanley et al (2016) demonstrate the potential for developing an institutional logics approach through their work researching a specific logic on participatory decision-making by examining drivers and barriers to individuals “doing democracy”. This study draws on a similar approach and adds an additional step using causation coding to determine the validity of identified dominant logics. Similarly, Saldaña (2016) suggests that to identify causality, it is necessary to look for clues beyond a linear cause and effect approach by looking for clues embedded in the data narratives through a deductive process. The method employed here draws on these insights by adopting a recursive approach to causal mapping by going back and forth between institutional concepts, emerging insights and ideas about causality from the analysis and the raw data from interviews.

## 2 Methods

I have selected a qualitative case study approach to understand the factors that shape how children participate in informal settlement revitalisation in Suva, Fiji. Qualitative research supports an abductive approach to grounding people’s social worldview and perspectives in theoretical foundations (Bryman 2012).

### 2.1 Selection of case study

This project focuses on the Revitalising Informal Settlements and their Environments (RISE) program as a case study. This project has been selected as it formally involves children’s participation and a diverse range of urban planning processes, and it fulfils the UN’s definition of a vulnerable setting, involving a small island state at risk of inundation due to the changing climate, informal settlements, high rates of poverty, and high disaster risk (Government of Fiji et al. 2017). Research in this region is also extremely limited. The RISE project is led by the Monash Sustainable Development Institute and is a randomised control research trial focused on health, environment, water and sanitation with impacts measured through monitoring of health and environmental outcomes. It is a complex project involving multiple organisations and funding partners which provides the opportunity for examining diverse perspectives. Its focus on codesign for the infrastructure design and the extraction of health and environmental samples in the field, all include some form of children’s participation, either planned or unplanned. The program includes urban planning processes such as negotiation and formalising of land tenure, site health analysis, codesign workshops, construction and management planning, drawing on a diverse range of disciplines including microbiology, ecology, population health, landscape architecture, architecture, construction, engineering, project management, water, sanitation and hygiene (WASH) and gender, and relies on the participation of all community members in different aspects of the program.

### 2.2 Data sources

A total of 32 interviews were conducted across RISE including 15 staff based in Fiji, 12 based in Australia and 5 based in USA with a gender split of 18 female and 14 male participants who consented as per the approved ethics process. Snowball sampling was used in the interview process until a point of saturation was reached where no new relevant information was being presented (Bryman 2012), and a broad cross-section of researchers and staff were selected from the program that represented the diversity of teams and program countries. Participants included the following disciplines and roles: RISE researchers, staff and public authority staff in the disciplines of design fields (such as engineering, landscape architecture, WASH), construction fields (including project management), public water authorities, ecology and environment, public health, health economics and gender studies.

### 2.3 Data analysis

Data analysis required an initial separation of the data into types of participation and identification of the factors that impacted on whether participation occurred and their impact on the types of participation that occurred. These two areas are described in more detail in sections 2.3.1 and 2.3.2.

#### 2.3.1 Characterising participation

Determining the factors that impact children’s participation in RISE first required the identification of whether children participated in any part of RISE, and how they participated which resulted in eight different types of children’s participation, including when participation did not occur. While the study does not evaluate the quality of children’s participation, identifying how they participate is critical for understanding the impact factors have on deciding to include or exclude children in different aspects of the urban planning process, the extent and frequency to which it occurred and whether children’s participation is embedded in any aspects of RISE or with individuals. Participation types are categorised against Pells’ terms ‘lived’ and ‘performed’ participation. Types are identified as sub-categories that are determined by the beneficial nature of the types of participation through interviewees’ explanations of what has occurred. The types of participation categories are as follows:

(1) Children as participants (children are subjects, participants, citizens, experts, agents)
  - ‘Performed’ participation
    - Extractive processes (e.g., Children viewed as objects of research and data such as health samples are taken from children)
    - Mutualistic processes (e.g., Children viewed as an interest group and workshops educate children as well as extract information from them)
    - Agentic processes (e.g., Children viewed as experts and agents and children have control over the format of a workshop)
  - ‘Lived’ participation
    - Extractive processes (e.g., Children look after the safety of RISE members with no obvious personal benefit)
    - Mutualistic processes (e.g., Children help RISE staff where staff require help in a mutually beneficial exchange)
    - Agentic processes (e.g., Children help RISE staff to collect field samples where staff do not require and have not asked for help)
(2) Children did not participate (children did not participate, children viewed as objects, exposure pathways, disease indicator/biological sentinels, sample, targets)
  - Extractive processes (e.g., Parents or adults participate on behalf of children)
  - Agentic processes (e.g., Children refuse to have health samples taken)

#### 2.3.2 Developing a children’s participation in RISE logic

The process for determining the factors that shape children’s participation in RISE commenced with codifying the entire pool of interview transcripts into 1614 text fragments against the research question: ‘What are the factors that shape how children participate in informal settlement revitalisation in Suva, Fiji?’ The resulting fragments were then categorised into the factors that impacted participation. This resulted in 205 factors that impacted participation.

Thornton, Ocasio and Lounsbury’s (2012) institutional logics framework has been adapted and used as a qualitative analytical lens to align factors with institutional characteristics. Dominant characteristics were determined followed by the impact they had on children’s participation. The final step resulted in the classification of RISE into a children’s participation logic recognising that within other areas of RISE, different logics may be influencing behaviours and decisions. Appendix 1 illustrates the original institutional framework. The additional characteristic of informal control mechanisms has been included from Thornton, Jones and Kury (2005) expanded framework. The resulting participation logic can be found in section 3.3 and is outlined as follows.

Column 1 outlines the characteristics or institutional carriers that represent the symbolic systems, tools, rituals, motivations, sense of self, activities and artifacts or objects that shape individuals’ and organisations’ behaviour (Thornton, Ocasio and Lounsbury 2012; Scott 2014; Thornton, Jones and Kury 2005).

Column 2 identifies the dominant institutional domain of each characteristic, noting that the logic with the strongest influence and impact on children’s participation is singled out for the purposes of how RISE as an institution operates at a high level towards children’s participation. An inductive approach was used to align factors and individual responses against each of the institutional categories and dominant logics determined through numbers and gaps. The use of numbers in this qualitative study simply demonstrates areas of dominance, complexity, and volume of thought processes and which institutional characteristics of RISE have the most significant impact on children’s participation.

Column 3 identifies whether the resulting behaviour from the institutional orders and characteristics supported children’s participation, or whether there were contradictory factors. Under the heading of main actors, multiple participatory behaviours are identified. This reflects the mixed logics within teams, and also the tension between team and individual logics.

An example of a contradictory factor can be understood through the example of interviewee #25 who consistently iterated that RISE was not the place for children’s participation or part of their role, but their actions resulted in children participating:

“I’m not sure if RISE is the platform for it [children’s participation]”.

The interviewee responses were listed as contradictory as they engaged with children through ‘lived agentic’ activities as per below:

“I noticed there were lots of kids and they were very interested in what we were doing and why we were there…and they were all kind of crowded around to see what I was doing, and they were really interested and engaged in that”.

Interviewee #21 further identified activities where they observed interviewee #25 involving children in their work. This example demonstrates that while interviewee #25 did not see their role as having a participatory component, they enabled children to participate so this is listed as a contradictory factor.

## 3 Results

### 3.1 Summary of results

In summary, the participatory logics of RISE are emerging into a mixed institutional logic, reflective of the diversity of professional disciplines, communities and settlements, and wide-ranging objectives in a large complex project. Overall, the state logic dominates children’s participation in RISE with corporation logic of secondary dominance. The state logic tended to support participation while the corporation logic did not support participation. Other institutional logics also impacted positively and negatively on children’s participation, however to a lesser degree.

While the results demonstrate that participation has occurred in various forms, the understanding of what participation could be, the purpose and the method for implementing a participatory approach is inconsistent and misunderstood. While RISE as a whole appears as a dominant set of participatory logics, the analysis of individual interviews demonstrated a multitude of tensions between competing logics and both internal and external factors. The desire to serve the participating communities in some form, however, presents as a potential willingness to explore individuals’ roles in embedding children’s participation into RISE in some form.

### 3.2 How do children participate in RISE Fiji?

Children’s participation varied across the teams in RISE both in a ‘performed’ capacity such as in workshops or health data extraction, and in a ‘lived’ capacity where participation occurred within daily life experiences based on Pells’ definitions (2010). To understand how children were participating across RISE I asked for examples of how children are participating in RISE, and whether children were participating in the participant’s work. Participants identified a range of ways that children participated, and where children did not participate.

The sections 3.1.1 and 3.1.2 follow Pells’ definitions of ‘performed’ and ‘lived’. Bolded terms are the sub-categories determined by this study.

#### 3.2.1 Performed participation

Participatory ‘codesign’ workshops were held to ensure members of households could understand and contribute to the development of the infrastructure. They were generally conducted by the teams tasked with designing and building infrastructure with support from the local water authority. Children’s participation varied considerably across the settlements but generally took the form of **mutualistic** processes where children participated in some workshops to support community education such as follows:

“We have to get the kids more involved because we want them to be able to go home and explain to their parents what they mean” – interviewee #6

In an example of agentic participation, children changed the format of a workshop which RISE staff allowed despite time restrictions:

*“We allowed them just to have that space and share their thoughts on the RISE system”* – interviewee #5 explained while unplanned, they allowed children to extend a workshop.

The success of RISE will be measured predominantly on the health and wellbeing of children under five years of age (RISE 2017b). This resulted in children participating in an **extractive** manner through collecting health samples directly from children. Interviewees expressed both empathy with children and recognition that obtaining samples from distressed participants was detrimental to the program such as the following statement:

“It’s just really important because folks have definitely refused to participate in an entire study based on whether or not a blood draw went well or if they perceived that it went well or perceived that it went poorly.” – interviewee #31

The decisions to include children in performed participatory activities were generally premised on the idea that participation is designed to extract data or ensure the success of the RISE interventions. The activities children participated in was dependent on whether children were identified upfront as essential in the planning phase of activities. How individuals viewed children’s role in these performed activities also impacted their direct interactions with children in those processes.

#### 3.2.2 Lived participation

Less understood or acknowledged as a form of participation were the examples of lived participation, mostly occurring as a direct result of children enacting their agency and RISE staff responding. In an example of **extractive** lived participation, children looked after the safety of RISE officers without obvious benefit to themselves:

*“I’ve had probably a 14-year-old, the fellow who asked - I actually asked him, ‘Can you just walk me back to the bus stop?’”* – interviewee #9 describing their fear of walking unattended through the settlements and asking for support from a child resident

In an example of **mutualistic** lived participation, interviewee #6 described how children identified a gap and offered to help RISE officers, offering their services as language translators which gave them a voluntary work opportunity:

"One of the youth from the community put their hand up and said, "Oh, I wouldn’t mind translating in Indo-Fijian", so in Hindi. None of the RISE staff spoke Hindi, so it was really good that the youth were putting their hands up for that." – interviewee #6.

While this service was well received, interviewees expressed that in future the preference would be to find an external translator.

In an example of **agentic** lived participation, children sought out RISE officers, demanding to participate or have their voices heard. Interviewee #9 in particular identified a number of instances where children exercised both their agency and their capability as demonstrated in the following example:

*“She…told me not to chop the mangroves down because what she learnt in school was that the mangroves stopped the waves coming in. She told me that…Then I had to justify - assure her that we were not going to be chopping mangroves.”* – interviewee #9 in this situation found themselves initially dismissing the child.

The ‘lived’ experiences identified in the RISE study challenge our understanding of participatory processes. The enabling responses from RISE staff and supporters opened up opportunities for mutually beneficial conversations or activities. Examples include RISE staff and supporters informally taking on the role of mentor and engaging in discussions about future career planning. These interactions also increased trust in the communities and support for the RISE intervention. A number of interviewees also identified possible approaches to overcoming issues of non-attendance by engaging in lived participation opportunities such as sharing food, which was identified as a strong Fijian cultural practice.

#### 3.2.3 No participation

The final category of participation is when children did not participate in parts of RISE.

In some of these cases, data was **extracted** from parents or caregivers. The following interviewee indicated that a clear decision was made for adults to participate on behalf of their children in different aspects of RISE.

"We’ve made the decision to go a parent proxy or caregiver proxy…the caregiver or the mother is usually the best person to talk to about that [health of children under 5]"- interviewee #16 describing why they chose to exclude children.

In the category of no participation children exhibited elements of **agentic** non-participation.

“I’ve seen that even though the parents consent for the child to participate, but some of them [children] are not comfortable, so they refuse to participate, and that’s okay.” – interviewee #11 describing children enacting their agency by refusing to participate.

Generally, the responses to children not participating were due to a planned approach to the extraction of data in the most efficient manner with beliefs that adults were able to provide what was needed. Other key factors included cultural factors such as the status of children in societal hierarchies, competing time constraints or children simply unwilling or unable to participate. There were some cases that demonstrated there had been limited to no discussion on the inclusion of children.

### 3.3 Institutional logics and impacts on children’s participation

This section presents the institutional analysis of children’s participation in RISE Fiji.

**Table:** 

Characteristic	RISE primary institutional logic (secondary logics shown in (). If no secondary logic noted, this indicates that there was a clear dominant logic)	Supports child participation Y/N/Contradictory
Root metaphor	*ST - State as redistribution mechanism (COM - Community common boundary)	Y (Y)
Mission	ST - Redistribution mechanism i.e. Create change through successful intervention - future outlook	C
Sources of legitimacy	ST - Democratic participation (PR - Personal expertise)	Y (Y)
Sources of authority	CO - Top management (FA - Patriarchal domination COM - Commitment to community values and ideology)	N (N) (Y)
Sources of identity	PR - Association with quality of craft (CO - Bureaucratic roles)	Y N
Basis of norms	ST - Citizen membership, Children are citizens in RISE communities - individual understandings of what this means	Y
Basis of attention	ST - Status of interest group - recognition that children are an interest group with individual and team understandings of what this means (FA - Status in household)	Y (N)
Basis of strategy	ST - General desire to increase community good with differing beliefs in how to achieve this (COM - increase status and honour of members and practices)	Y (Y)
Informal Control Mechanisms	CO - Organisation culture - mechanisms vary according to sub-organisations of RISE	C
Formal control mechanisms/Practices (types and forms of participation)	CO - Board of management authority overseeing formal systems of co-design, taking of samples, extractive `performed' participatory processes, etc. (ST - Enforcement of legislation/regulative processes)	C (N)
Main actors	RISE researchers, staff, public authority staff in the disciplines of design fields (such as engineering, landscape architecture, WASH), construction fields (including project management), public water authorities, ecology and environment, public health, health economics, gender studies	Y/N/C (variable across teams)
Economic system	CO - Managerial capitalism - role and impact of management structures have a strong bearing on participatory practices (COM- cooperative capitalism - cultural impact of shared practices)	N (Y)
Funding	CO/ST - Global foundation, bank (corporation), INGOs, governments, universities	C

* Logic abbreviations: FA =Family, COM = Community, ST = State, PR = Profession, CO = Corporation

This section demonstrates how the overall logics were determined through the dominant characteristics identified in the interviews. Characteristics are highlighted in **bold** for easy reference back to the table and follow the order listed in the table. The **root metaphor** or narrative of RISE articulated by interviewees is that of a redistribution mechanism for funding and research. RISE’s **mission** is to deliver a research-based approach underpinned by the discipline of ‘planetary health’, through a ‘localised water-sensitive approach to revitalising informal settlements’ (RISE 2017b). The following interviewee expressed their understanding of the **mission** impacting across these levels either highlighting specifically impacted population groups through to higher level areas of impact:

“We are not just trying to change the environment because we think it would be nice to change the environment. We are making the argument that there is a connection between environment and health and that is going to go through to the children. I see this as something that is, kind of, central to our whole rationale, our whole storyline.” – interview #28

**Sources of legitimacy** align with the state domain where individuals and teams recognised children as democratic participants. Thornton, Ocasio and Lounsbury (2012) restricts their definition of community to the boundaries of specific interpersonal relationships or specific group membership. Where interviewees demonstrated a care for all including children, the factors they identified were aligned with a state logic (see Appendix 1).

Interviewee #26 articulated the role of children as recognised citizens in the program:

"We're recognising that a community is made up of children and adults, and our whole approach, I think, is - spans that spectrum".

The structure of the RISE program is largely aligned with a corporate logic, with a hierarchical management structure as the **key source of authority** and bureaucratic roles as interviewees’ **source of identity**. Some interviewees indicated that only tasks directly aligned with their roles were conducted. This largely resulted in children’s participation being restricted to those roles that were directly responsible activities where children’s participation was explicit.

*“Obviously because my role in RISE is across program, I’m not the one leading those activities”* – interviewee #24 demonstrated a strong belief in children’s participation, yet this did not translate into action because of their role.

Views on interviewees’ **source of authority** were conflicted. The following statement demonstrated leadership was influential, however did not translate into mainstreamed participation:

“We’re thankful that we have a good boss who really emphasize our children’s participation in our community.” – interviewee #4 describing the influence of their manager for encouraging children’s participation.

Interviewees’ **source of identity** strongly connected to profession logic characteristic associated with the quality of their craft, sometimes contradicting interviewees’ identification with their role. The profession logic manifested as equating quality with success of the intervention necessitating a participatory approach. Interviewee #20 noted that:

“And we need to make sure that we do hear from everyone, so that we don’t have someone at the end coming back and saying, “actually, you’ve made my life harder, because you’ve put a septic tank where I used to access the mosque, or the church, and now I can’t walk that way.” And to me, success is that we don’t have anyone feel like that.”

For many interviewees, their **basis of norms** and **attention** recognised children as key participants of RISE with a focus on their status within RISE.

“In a cultural setting we are so mindful of the future, of the village, of the future of the community, that is why we try to upskill these children, upskill their knowledge and build their capacity because they are the future of the communities, they are the future of their village.” – interviewee #8 demonstrating the importance of children’s participation as future caretakers.

A general **basis of strategy** demonstrated a genuine desire to increase community good and a recognition that children are members of the communities. The following interviewees demonstrated their commitment to improving community health and wellbeing:

“So just the little that we can do for these communities I think is what drives us in this project, especially to help children. Or help those that are a very young age as they grow up.” – interviewee #4 described their strategy for improving conditions for children.

**Informal and formal control mechanisms** included workshops, processes for extracting samples, ethics processes, training, budgeting, and policies. Generally, these systems were either narrow and specific in their focus, seen as restrictive, or poorly understood. The following interviewee described their reluctance to engage with children in any form due to the strict protocols in place:

“There’s no direct interaction with them. It’s because of the policies that are in place.” – interviewee #13.

Participatory elements such as ‘co-design’ were identified in the funding application to the Wellcome Trust and translated into RISE objectives^ii^. However, this didn’t automatically result in children’s participation:

“The funders – and I’ve been having this conversation a lot lately it feels like, because funders expect stakeholder engagement, but they don’t actually – they don’t allow for time or funding or resources, capacity.” – interviewee #32 described their frustration at limited resourcing from funding bodies.

Perhaps the greatest impact on children’s participation in workshops, however, was that children were simply not explicitly invited.

“When we initially sent out the invite and informed communities, we had encouraged the participation of heads of households. And I guess that’s what the community had stuck with when coming to the workshops, that it was only meant for adults” – interviewee #5

In summary, categories within a state order generally translated into the occurrence of children’s participation with corporate logics hindering participation. There was a recognition that children are critical citizens in the RISE program; and as the program progresses and the value of children’s participation emerges, there is a genuine recognition and desire to increase children’s participation.

#### 3.3.3 Impact of other institutional logics

Market and religion logics barely rated as impacting participation. Market logics were noted as a transactional **root metaphor** or a **basis of strategy** to increase efficiency, both of which negatively impacted participation. References to religious impact were negligible. Community, profession, and family logics had some bearing on children’s participation in RISE.

The characteristics in community logic that supported participation were apparent when interviewees’ **source of authority** and their **basis of attention** was focused on their personal investment in the group, and they believed in an **economic system** of cooperative capitalism demonstrated by interviewee #7’s comments:

“We are committed and invested in communities… Fijians, we are communal people. Everything is shared, so it’s like a muscle memory; we don’t think about sharing… I think it’s engrained. It’s engrained in our being, in our culture, to share. So, there are always people who are sharing, we pride in - our wealth is in our sharing.” – interviewee #7

Where community and state logics worked together, children’s participation was strongest in RISE, however there was a continued tension with corporate logic characteristics of **sources of authority, informal and formal control mechanisms** such as organisational practices.

Family logics sometimes overrode other logics and resulted in children not participating due to patriarchal domination as the **source of authority** and the **basis of attention** on children’s status within the household. Family logics of patriarchal domination and the status of children in the household varied across the 12 settlements and between different ethnic groups. While this was considered a key barrier, there was also a belief that this could be overcome if the purposes of children’s participation were communicated well.

## 4 Discussion

### 4.1 How do institutional logics impact children’s participation in RISE?

The results demonstrate that the factors that impact children’s participation are more complex than general causes outlined in the UN documents. The institutional logics model provides a deconstructed view of the structures and processes that influence children’s participation in RISE. They demonstrate not only the cascading impact of individual characteristics within the logics model, but also the powerful impact individuals can have on the inclusion or exclusion of children regardless of the dominant logics of the organisations they work in. This is supported by Thornton, Ocasio and Lounsbury’s (2012, 179) assertion that individuals can draw on multiple and competing institutional orders.

Strong personal motivations demonstrated the complexity of institutional logics at play, and the way individuals perceive children. Generally, children’s participation was perceived by participants to be a resource-intensive, formally constructed approach to extracting data, however there were numerous examples of children participating as a result of their own direct intervention into the program, enabled by individuals. The acceptance of children’s agency was largely dependent on individual alignment with particular institutional logics, and the value of their agency was only partly recognised. Children’s agency was further strengthened when a team shared a focus on all interest groups including children. Holloway, Holt and Mills (2019) support these assertions by suggesting that children’s ability to enact their agency is interdependent on their social environment.

Interviewees expressed assumptions as to how children’s participation could be better supported, however the logics model demonstrates that individual institutional characteristics are inadequate on their own for engendering an embedded culture of children’s participation. For instance, the development of a RISE participatory toolkit and framework may go some way towards embedding participation which may act as an informal control mechanism. However, a number of authors identify tools on their own are insufficient for embedding children’s participation and should be part of a suite of transformative interventions (Cele and Van Der Burgt 2015; Nordström and Wales 2019; Freeman and Aitken-Rose 2005).

RISE is evolving as a program and the institutional logics of RISE are still emerging. Thornton, Ocasio and Lounsbury (2012) suggest that this type of instability actually supports the opportunity for transformation. They suggest that change can occur through altering existing theories, frames and narratives to influence strengthening of existing practices and creating new vocabularies of practice and behaviours (Thornton, Ocasio and Lounsbury 2012, 161). This research study can help determine how RISE strengthens children’s participation going forward. This will involve determining what types of participation RISE will support, what impact they hope to achieve through participation and for whom, and their level of commitment for transformative change.

### 4.2 Implications for contextually and place-specific practices for children’s participation

The development of a typology for children’s participation forces urban planners to examine their motivations for including or excluding children and expands understanding of participation. The results demonstrate that children’s participation is misunderstood, adult-led and generally extractive in nature even when well-intentioned and mutualistic. The research study was limited as it did not capture the views of children. This hinders our ability to understand the value and impact of participatory processes on children, types of participation that the study may have missed, and types of participation preferred by children that did not occur. This research study also did not capture the views of household adults responsible for children such as parents which limits our understanding of the level of control household adults have over children’s participation.

The United Nations Committee on the Rights of the Child defines children’s participation as either ‘tokenistic’ or ‘meaningful’, cautioning States against the dangers of ‘tokenistic’ efforts (UN Committee on the Rights of the Child (CRC) 2009). While the damage of poor participatory activities is real (Mansfield, Batagol and Raven 2021), the judgement of what constitutes meaningful processes is at risk of being determined by adults, irrespective of place- and cultural-specific conditions. Labelling participation as ‘tokenistic’ or ‘meaningful’ also presupposes a ‘performed’ series of activities designed to extract information from children (Malone and Hartung 2010; Pells 2010). The downside of such an approach is the inference that time and resources need to be factored into projects as separate items that can then be influenced, and that the participatory process itself is a finite series of events. Liebel and Saadi (2009) further comment that viewing children’s participation through the lens of different cultures, can also be understood through children’s contribution to their community via the undertaking of economic, political and social tasks. This assertion was supported by the references to children’s role in Fijian culture, which varied between settlements, serving as a reminder that children cannot be treated as a homogenous group when planning participatory approaches.

Place- and cultural-specific issues require a rethink of approaches to participation based on the localised understanding of participation, social and cultural impacts of children’s participation, and the burden on communities to participate in activities outside their normal routines. This suggests that a greater upfront understanding of locally specific activities, may present opportunities to join in existing activities, rather than developing separate, extra activities that present a burden in already stretched communities (Derr and Tarantini 2016).

Perhaps the most important challenge is to better understand how to be invited into and meet children safely in their domain to engage in lived participation. In this way children can exercise their agency in a protected, supportive, culturally, and socially receptive environment. Some individuals appear to do this naturally yet without understanding their impact. These individuals may have the potential to support embedding a culture of participation in organisations as an ally, supported by Matthews’ and Limb’s (1999) assertion that allies are the way for children to gain entry into adults’ world.

The identification of a typology of participation is an important step towards understanding participation, how it impacts children and how urban planners may perceive their own role when conducting participatory approaches. The typology questions the role of an urban planner as a receiver of information pertaining to their project or process. It serves as a reminder that their very presence has impact with potential long-term benefits such as influencing career aspirations of participating children. Understanding participation through a cultural and local lens is critical for determining appropriate methods and learning how children already participate within their social, cultural, and familial settings. The added burden of performed participatory activities runs the risk of further exclusion of children and can impact important social systems. While the institutional logics model provides some guidance on how children’s participation may be improved or embedded within an organization, urban planners need to understand the local external and social logics to ensure participation is meaningful and does not work against local systems.

### 4.3 Implications for urban planning fields working in informal settlements

This study provides guidance for transforming urban planning individuals, organisations and discipline behaviours, practices, and the resource environment through a shifting of institutional logics to support children’s participation in urban planning processes in informal settlements. It also demonstrates that participation is likely to be ad hoc unless it is embedded through institutional logics that recognize children as active, willing, and capable citizens in their settlements. Thornton, Jones and Kury (2005) and Thornton, Ocasio and Lounsbury (2012) demonstrate that conflict and contradictions provide the environment to mobilise resources and develop institutional change at the field level.

This study also serves as a cautionary warning. It is critical that urban planners understand local socio-cultural practices, hierarchies, and beliefs before embarking on a child-participatory process, not only for successful processes to occur, but also to ensure that no harm is done to participating communities. Ultimately the success of mainstreaming children’s participation in urban planning processes for informal settlements is reliant on taking this research a step further to understand the cultural systems that the institutional logics sit within. This requires deep commitment from urban planners to address the logics that perpetuate a culture of children’s exclusion.

## 5 Conclusion

This study provides valuable lessons for urban planners working in informal settlements. Urban planners have a responsibility to ensure that policy and planning processes are participatory to improve living conditions in settlements and successfully deliver on SDG target 11.3. This study set out to answer the research question ‘What are the factors that shape how children participate in informal settlement revitalisation in Suva, Fiji?’ Two key dimensions arise from this study. First, the results demonstrated that limited understanding of the purpose and types of participation was a key factor that impacted how and whether participation occurred and in what parts of RISE. Secondly, the institutional logics model demonstrates that there is a myriad of complex, intertwined factors that sit at societal, organizational, and individual levels that often contradict each other and perpetuate exclusionary practices.

While the institutional logics model helps in understanding the complexity of the barriers and enablers, challenges remain. The logics model does not sit in isolation but is influenced by and linked to societal and external logics, theories, frames, narratives, vocabularies of practice, resource environments and practice, all of which contribute to the broader culture of children’s participation. These other aspects need to be understood before determining conclusive action for developing an embedded culture of children’s participation in any urban planning processes in informal settlements. Ultimately, the research demonstrates that the United Nations’ call to address high-level barriers to children’s participation requires urban planners to develop a more nuanced approach to embed a culture of children’s participation in urban planning in informal settlements.

## Supplementary Material

Appendix 1

## Footnotes

i RISE is an action-research program. The program is underpinned by the discipline of ’Planetary Health’ and has developed a randomised-control-trial for developing localised, water-sensitive approaches to revitalising informal settlements. The program is working with communities, governments, local leaders and partner institutions using a co-design inclusive approach across 24 informal settlements in Suva, Fiji and Makassar, Indonesia RISE. 2017b.

ii The RISE objectives as listed in the 2017 Annual Activity Report include: “Objective 1 ‘design and engagement: implementation of the WSC [water-sensitive cities] revitalisation of urban informal settlements through co-design processes, reflecting community aspirations and site contexts’; Objective 2 ‘ecology and environment: environmental monitoring will determine the impact of the intervention on the prevalence and density of microbial communities and faecal pathogens, biodiversity, and vector abundance; Objective 3 ‘human health: a crucial hypothesis is that the environmental benefits of the intervention are accompanied by health improvements. The impact of the altered environment on the health of residents is being assessed, prioritising biological evaluation of gastrointestinal health of children under five years of age; Objective 4 ‘well-being: the physical environment is a significant structural determinant of well-being. Changes to this environment can affect how people live, how they feel about themselves and their lives, and how safe they feel. It can affect an individual’s capacity for paid work and to feel part of a community. Objective 4 will monitor the effects of the intervention on individual and community well-being; and Objective 5 Policy and investment: Objective 5 will integrate evidence and outcomes from across RISE to facilitate the widespread adoption of a WSC approach to revitalisation of informal settlements. The aim is to facilitate transferability of program lessons learned and outcomes to end users, including government, NGOs, communities, professionals and the private sector who shape urban development and water management decisions and practices.” - RISE 2017a. Laying Foundations - RISE Annual Activity Report 2017. RISE.

## References

[R1] Bryman A (2012). Social Research Methods.

[R2] Cele S, Van Der Burgt D (2015). Participation, Consultation, Confusion: Professionals' Understandings of Children's Participation in Physical Planning. Children's Geographies.

[R3] Derr V, Tarantini E (2016). “Because We Are All People”: Outcomes and Reflections from Young People’s Participation in the Planning and Design of Child-Friendly Public Spaces. Local Environment.

[R4] Government of Fiji, World Bank, GFFDRR & ACP-EU (2017). Climate Vulnerability Assessment - Making Fiji Climate Resilient.

[R5] Freeman C, Aitken-Rose E (2005). Voices Of Youth: Planning Projects with Children and Youth People in New Zealand Local Government. Town Planning Review.

[R6] Fuenfschilling L, Truffer B (2014). The Structuration of Socio-Technical Regimes - Conceptual Foundations from Institutional Theory. Research policy.

[R7] Hart RA (1997). Children's Participation: The Theory and Practice Of Involving Young Citizens in Community Development and Environmental Care.

[R8] Hart RA, Reid A, Jensen BB, Nikel J, Simovska V (2008). Participation and Learning: Developing Perspectives on Education and the Environment, Health and Sustainability.

[R9] Holloway S, Holt L, Mills S (2019). Questions Of Agency: Capacity, Subjectivity, Spatiality and Temporality. Progress in Human Geography.

[R10] Liebel M, Saadi I, Percy-Smith B, Thomas N (2009). A Handbook of Children and Young People's Participation: Perspectives from Theory and Practice.

[R11] Malone K, Hartung C, Percy-Smith B, Thomas N (2010). A Handbook of Children and Young People's Participation: Perspectives from Theory and Practice.

[R12] Mansfield RG, Batagol B, Raven R (2021). “Critical Agents of Change?”: Opportunities and Limits to Children’s Participation in Urban Planning. Journal of planning literature.

[R13] Mason J, Bolzan N, Percy-Smith B, Thomas N (2009). A Handbook of Children And Young People’s Participation: Perspectives from Theory And Practice.

[R14] Matthews H, Limb M (1999). Defining an Agenda for the Geography of Children: Review And Prospect. Progress in Human Geography.

[R15] Nordström M, Wales M (2019). Enhancing Urban Transformative Capacity Through Children's Participation in Planning. Ambio.

[R16] Pells K, Percy-Smith B, Thomas N (2010). A Handbook of Children and Young People’s Participation: Perspectives from Theory And Practice.

[R17] Reay T, Jones C (2016). Qualitatively Capturing Institutional Logics. Strategic Organization.

[R18] Rise (2017a). Laying Foundations - RISE Annual Activity Report 2017.

[R19] Rise (2017b). Revitalising Informal Settlements and their Environments.

[R20] Saldaña J (2016). The Coding Manual for Qualitative Researchers.

[R21] Scott WR (2014). Institutions and Organizations: Ideas, Interests and Identities.

[R22] Thornton PH, Jones C, Kury K (2005). Transformation in Cultural Industries.

[R23] Thornton PH, Ocasio W, Lounsbury M (2012). The Institutional Logics Perspective: A New Approach to Culture, Structure and Process.

[R24] UN Committee on the Rights of the Child (CRC) (2009). General Comment No.12 (2009): The Right of the Child to be Heard.

[R25] UN General Assembly (1989). Convention on the Rights of the Child.

[R26] UN General Assembly (2002). Resolution adopted by the General Assembly on 11 October 2002: A/RES/S-27/2: A World Fit for Children.

[R27] UN General Assembly (2017a). Resolution adopted by the General Assembly on 6 July 2017: A/RES/71/313 Work of the Statistical Commission pertaining to the 2030 Agenda for Sustainable Development.

[R28] UN General Assembly (2017b). Resolution adopted by the General Assembly on 30 January 2017: A/RES71/177 Rights of the child.

[R29] UN General Assembly (2019). Resolution adopted by the General Assembly on 15 October 2019: A/RES/74/4 Political declaration of the high-level political forum on sustainable development convened under the auspices of the General Assembly.

[R30] UN General Assembly (2016). Resolution adopted by the General Assembly on 23 December 2016. A/RES/71/256* New Urban Agenda.

[R31] UN Secretary General (2019). Report of the Secretary-General on Progress Towards the Sustainable Development Goals (Special Edition) (E/2019/68).

[R32] UN Secretary General (2020). Report of the Secretary-General on Progress Towards the Sustainable Development Goals (Special Edition) (E/2020/57).

[R33] UN Secretary General (2021). Report of the Secretary-General on Progress Towards the Sustainable Development Goals (E/2021/58).

[R34] United Nations (2021). Status of Treaties United Nations Treaty Collection.

[R35] Winstanley A, Ahuriri-Driscoll A, Hepi M, Baker V, Foote J (2016). Understanding the Impact of Democratic Logics on Participatory Resource Decision-Making In New Zealand. Local environment.

