# Supplementary material for: “They’ll be the ones that’s looking after it” - Unravelling institutional factors that shape children’s participation in urban planning for informal settlements
Source: Child Geogr. Author manuscript; Available in PMC 2025 Nov 30. (PMC7618419; doi:10.1080/14733285.2022.2159331)

© Thornton et al’s Revised Interinstitutional System Ideal Types (Thornton et al., 2012). Reproduced with permission of the Licensor through PLSclear.


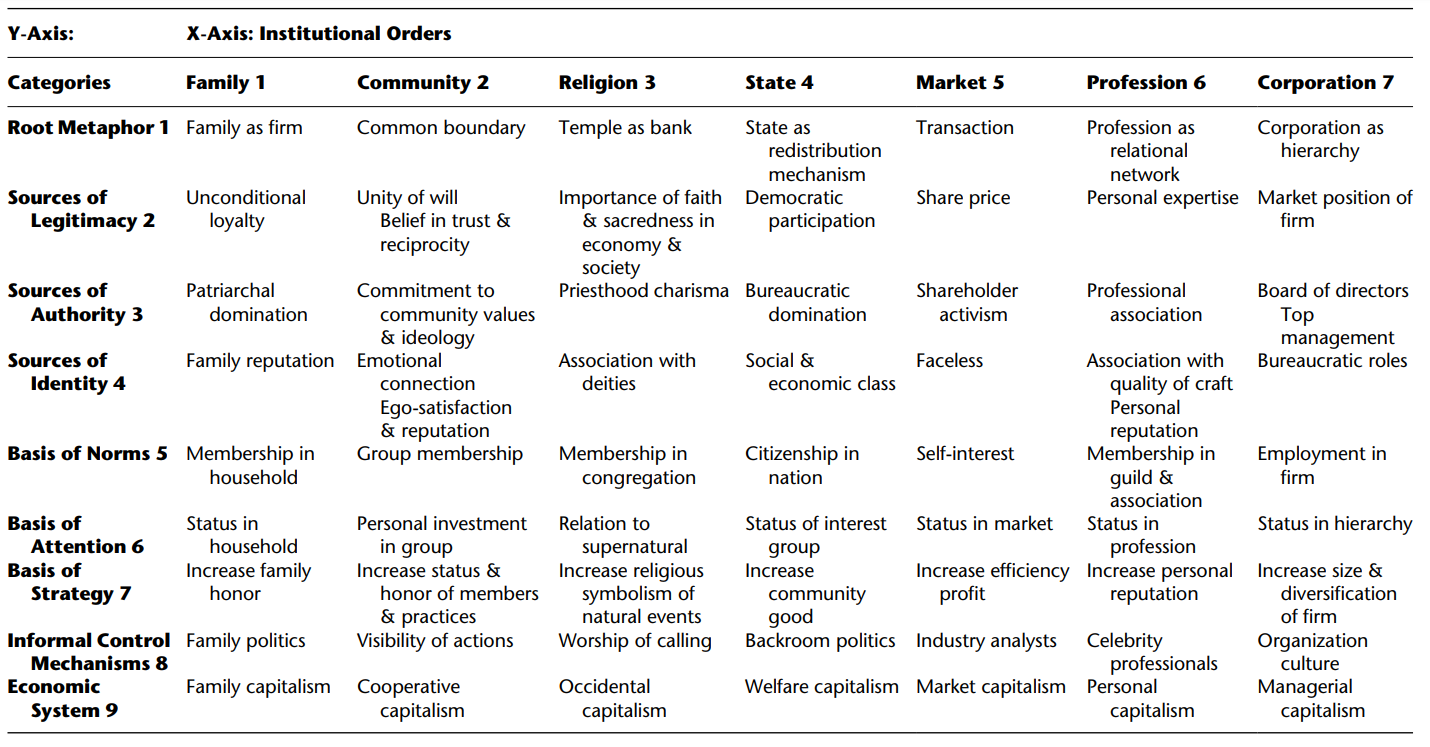

Supplement: Appendix 1 [file EMS210582-supplement-Appendix_1.docx]
